# Comparative Metagenomic Study of Rhizospheric and Bulk Mercury-Contaminated Soils in the Mining District of Almadén

**DOI:** 10.3389/fmicb.2022.797444

**Published:** 2022-03-07

**Authors:** Daniel González, Marina Robas, Vanesa Fernández, Marta Bárcena, Agustín Probanza, Pedro A. Jiménez

**Affiliations:** Department of Pharmaceutical Science and Health, CEU Universities, Boadilla del Monte, Spain

**Keywords:** PGPR, antibiotics, bioremediation, shotgun metagenomics, co-selection

## Abstract

Soil contamination by heavy metals, particularly mercury (Hg), is a problem that can seriously affect the environment, animals, and human health. Hg has the capacity to biomagnify in the food chain. That fact can lead to pathologies, of those which affect the central nervous system being the most severe. It is convenient to know the biological environmental indicators that alert of the effects of Hg contamination as well as the biological mechanisms that can help in its remediation. To contribute to this knowledge, this study conducted comparative analysis by the use of Shotgun metagenomics of the microbial communities in rhizospheric soils and bulk soil of the mining region of Almadén (Ciudad Real, Spain), one of the most affected areas by Hg in the world The sequences obtained was analyzed with MetaPhlAn2 tool and SUPER-FOCUS. The most abundant taxa in the taxonomic analysis in bulk soil were those of Actinobateria and Alphaproteobacteria. On the contrary, in the rhizospheric soil microorganisms belonging to the phylum Proteobacteria were abundant, evidencing that roots have a selective effect on the rhizospheric communities. In order to analyze possible indicators of biological contamination, a functional potential analysis was performed. The results point to a co-selection of the mechanisms of resistance to Hg and the mechanisms of resistance to antibiotics or other toxic compounds in environments contaminated by Hg. Likewise, the finding of antibiotic resistance mechanisms typical of the human clinic, such as resistance to beta-lactams and glycopeptics (vancomycin), suggests that these environments can behave as reservoirs. The sequences involved in Hg resistance (operon mer and efflux pumps) have a similar abundance in both soil types. However, the response to abiotic stress (salinity, desiccation, and contaminants) is more prevalent in rhizospheric soil. Finally, sequences involved in nitrogen fixation and metabolism and plant growth promotion (PGP genes) were identified, with higher relative abundances in rhizospheric soils. These findings can be the starting point for the targeted search for microorganisms suitable for further use in bioremediation processes in Hg-contaminated environments.

## Introduction

Mercury (Hg) is a highly toxic element that severely affects ecosystems (Hsu-Kim et al., 2018; Liu et al., 2018). It has the capacity to enter and biomagnify in the food chain and therefore affects human health even at low concentrations (Bjørklund et al., 2019). The accumulation of Hg can lead to pathologies, with those affecting the central nervous system being the most serious, such as Minamata syndrome (Gil-Hernández et al., 2020; Marumoto et al., 2020). The presence of Hg in various ecosystems is widely described. Exceptionally, environments with extremely high concentrations of this heavy metal have been described, such as those detected in the Almadén mercury mining region (> 8889 μg/g) (US Environmental Protection Agency, 2011).

The presence of Hg in soils conditions the development of organisms that inhabit it, with bacterial communities being one of the most vulnerable groups. Some of the bacterial species capable of resisting the presence of this pollutant could be suitable in processes of remediating affected soils, this is why there is a growing scientific interest in knowing the composition of these Hg-tolerant edaphic communities (Zhao et al., 2021). There are several references to the usefulness of these techniques in soil samples (Li et al., 2018; Westmann et al., 2018; Castillo Villamizar et al., 2019; Nelkner et al., 2019) and, in particular, in soils contaminated with different toxins (Garrido-Sanz et al., 2018; Kumar et al., 2018; Thomas et al., 2019).

Metagenomics consist of the complete study of genetic material extracted from a sample. Various metagenomic methods based on either DNA amplification and sequencing or DNA fragmentation and alignment are currently available (Giagnoni et al., 2018). One of the main metagenomic techniques, based on sequencing, is the creation of genetic libraries. The bioinformatic analysis of the data obtained allows us to reconstruct the metabolism of the organisms that make up the community and to predict their potential functional roles in the ecosystem through the so-called “environmental gene labels” (Youngblut et al., 2020). This field has also been called environmental genomics, ecogenomics or community genomics (Riesenfeld et al., 2004; Cordier et al., 2021). Methods based on functional analysis of DNA libraries from the entire microbial community of a particular medium can be a great source for the discovery of new genes (Handelsman, 2004; Douglas et al., 2020; Moon et al., 2020), study of unculturable microorganisms (Handelsman, 2004; Tyson et al., 2004; Cycil et al., 2020) and creation of genomic libraries (Enagbonma et al., 2020). This approach has also been used successfully in the study of antibiotic resistance in complex communities (de Abreu et al., 2020). One of the most widely used methods of study in metagenomics, and used in this work, is the “Shotgun metagenomics” technique. It consists of purifying the sample’s DNA and randomly fragmenting it into many small sequences that align into consensus sequences. These sequences are processed through analysis programs that allow taxonomic and functional identification of the sample’s DNA. SUPER-FOCUS is a bioinformatic tool which use the non-annotated sequences to predict potential functional activity, this allows study the whole metagenome as one unit and reveling the functional potential profile of the whole community (Chacón-Vargas et al., 2020; Collins et al., 2021).

To this end, this study aims to: (1) Find the taxonomic proportion and composition of the microbiological community of the soils of Almadén. (2) It seeks to provide an interpretation of the ecological behavior of the community, analyzing its functional potential information with SUPER-FOCUS.

## Materials and Methods

### Study and Sampling Area

The samples analyzed came from the mining district of Almadén, Ciudad Real (Spain), and were collected during the spring season Specifically, the slope “S” of Cerro Buitrones was sampled from the so-called “Plot 6” (38°46′25.1″N 4°51′03.9″W), described by other authors in previous studies (Millán et al., 2007). The concentration of Hg in this plot was 1710 mg/kg total Hg, 0.609 mg/kg soluble Hg and 7.3 mg/kg exchangeable Hg. Soil samples for the metagenomic study were obtained from the rhizosphere and bulk soil, together with plants described by Robas et al. (2021a): *Rumex induratus* Boiss. and Reut., *Rumex bucephalophorus* L., *Avena sativa* L., *Medicago sativa* L. and *Vicia benghalensis* L. (Robas et al., 2021a).

#### Production of Soil

To obtain samples of rhizospheric soil (RS), the root of each plant specimen was gently shaken in order to remove soil fractions that were not tightly adhering to the root. The part of the soil attached to the root was then carefully separated to make up 2 g per plant. The five rhizospheric fractions were then combined into a single sample, in order to obtain10 g of soil that was homogenized for further metagenomic study. The 10 g of bulk soil (BS) was obtained in the same way, by sampling 2 g of soil near each plant, avoiding the rhizospheric fraction. Each sample was divided into 3 technical replicates for the metagenomic analysis.

#### Isolation of DNA

The DNA was purified by the “DNeasy Power Soil Pro Kit” (Qiagen, United States) following the manufacturer’s instructions. An enzyme lysis step with lysozyme was included in order to obtain the highest and best amount of total bacterial DNA. Purified DNA was quantified using PicoGreenTM (Thermo Fisher Scientific, United States) from 40 pg. The genetic libraries were constructed using mechanical fragmentation and adaptors ligation by TruSeq (Illumina ^®^, United States) methodology. The metagenome sequences obtained were assembled using metaSPAdes tool (Nurk et al., 2017).

### Metagenomic and Bioinformatics Analysis

DNA isolated from BS and RS samples was used for metagenomic analysis. These samples were processed and sequenced with Shotgun using Illumina ^®^ MiSeq desktop using the 2 × 250 bp paired-end reagent V2 Kits (Illumina ^®^, United States) technology with a standard quantification pattern. Bioinformatic analysis and quality control were performed using the Fast QC tool (Andrews, 2017). Q-score was used to predict the probability of an error in base-calling. Over 75% of bases > Q30 averaged across the entire run was considered acceptable. Raw sequence reads underwent quality trimming using Trimmomatic to remove adaptor contaminants and low-quality reads (Li et al., 2010).

#### Taxonomic Analysis

The MetaPhlAn2 (Metagenomic Phylogenetic Analysis v2) tool was used for taxonomic analysis (Segata et al., 2012; Truong et al., 2015). This is a computational tool for profiling the composition of microbial communities from metagenomic shotgun sequencing data. MetaPhlAn relies on unique clade-specific marker genes identified from ∼17,000 reference genomes (∼13,500 bacterial and archaeal, ∼3,500 viral, and ∼110 eukaryotic) to make taxonomic predictions. It was used bowtie2 –bt2_ps “very-sensitive” preset parameters, –tax_lev “a” for prediction of all taxonomic levels, “–min_cu_len 2000” for minimum total nucleotide length for the markers in the clade, and “–stat_q 0,1” for quantile value.

#### Functional Analysis

The SUPER–FOCUS tool (SUbsystems Profile by databasE Reduction) was used for the functional potential analysis of the data obtained by Shotgun metagenomics. FOCUS uses a reference database to identify subsystems (predicted protein groups with similar potential function) (Silva et al., 2016). This tool reports functional annotation using CD-HIT and with the SEED database, we reduced the references of the data set (Overbeek et al., 2004; Aziz et al., 2012). SUPER-FOCUS identifies the taxonomic profile of the data and creates a database with the subsystems for predicted organism. Metagenomic data was aligned against the database using RAPSearch2 (Zhao et al., 2012). Sequences with e-values ≤ 1e-5, a minimum identity of 60%, and an alignment length ≥ 15 amino acids were retained. This database categorizes information into three levels of detail: “level I” (large functional potential groups), “level II” (families of potential functional activities) and “level III” and SEED (specific potential functional role and the protein to which the sequences belong) (Silva et al., 2016).

#### Statistical Analysis

For the statistical analysis, SPSS v.27.0 program (Version 27.0 IBM Corp, Armonk, NY, United States) was used. In order to evaluate the quality of the technical replicates in each soil a Pearson correlation (r) of the percent genus abundances was done. Simpson and Shannon diversity index were also calculated with the relative abundances obtained from the taxonomical analysis to assess the ecological richness between BS and RS.

## Results

In the metagenomic DNA extraction and sequencing of RS and BS samples, were generated a total of 15,939,287 and 15,826,564 raw reads across the three technical replicates respectively, maintaining the 98.1% (RS) and 95.3% (BS) of the sequences after QC. The sequences were aligned and analyzed in two steps, taxonomical identification and functional annotation of the sequences. Species abundance between technical replicates was highly correlated (all comparisons *r* > 0.99 with Pearson correlation test). Processing a larger number of samples would allow obtaining statistically more complete information and reducing the limitation of the results in subsequent studies. The following results are presented divide by taxonomic identification and functional potential analysis.

### Taxonomic Identification

Taxonomic profile and relative abundances of the microbial community in RS and RF was analyzed using MetaPhlAn2. The identification of organisms is based on the assignment of the gene pool to a taxon. Comparing the BS and RS samples, a difference in abundance between viruses and bacteria was observed with an apparent 21 and 79% relative abundance respectively in RS and a 2 and 98% relative abundance respectively in BS.

**FIGURE 1:**
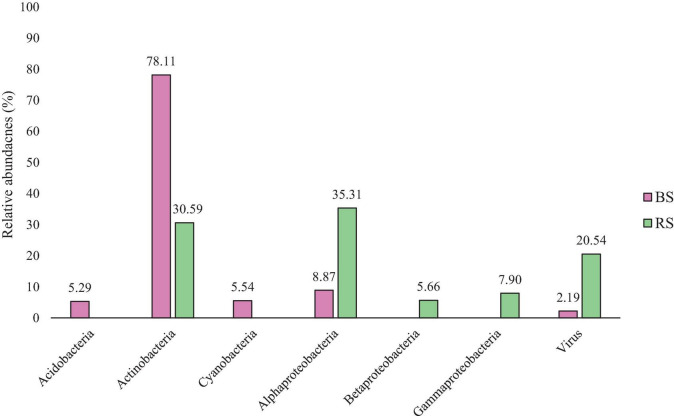
Comparative of the Relative abundances by taxonomic level of Class in BS (Pink - Bulk Soil) and RS (Green - Rhizospheric Soil). Data in the figure show how the relative abundances of the diferent classes are further grouped according to their presence in RS or BS.

Figure 1 shows the relative abundances of different bacterial groups. Examining the results obtained in BS, shows that the most abundant group is Actinobacteria. However, the best represented taxon in RS is Alpharoteobacteria. Acidobacteria and Cyanobacteria only appear in BS, and Betaproteobacteria and Gammaproteobacteria are only represented in RS.

**FIGURE 2:**
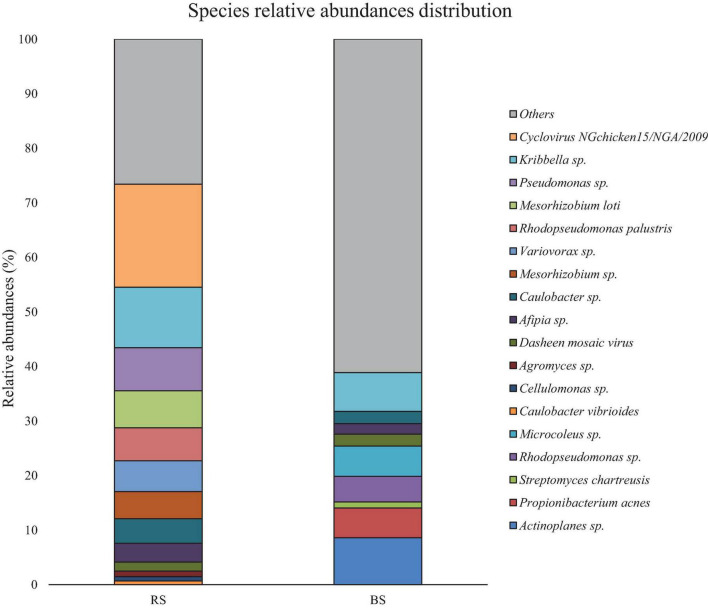
Comparative of the population relative abundances by taxonomic level of species in RS and BS. A pool of species could not be identifided with the sequences present in the metagenome in both soil.

Figure 2 shows the results of the relative abundances for the species taxon. Both samples seem to have a high diversity levels [Simpson’s diversity (D) and Shannon’s diversity (H)], being RS diversity levels (*D_*RS*_* = 0.14, *H_*RS*_* = 9.18) higher than BS (*D_*BS*_* = 0.4, *H_*BS*_* = 4.3). In RS, 73.42% of the genetic material was identified, leaving 26.58% unidentified (Figure 2 and Supplementary Table 1). Similarly, in BS 38.87% were identified. 61.13% of this DNA belongs to the various taxa (Figure 2 and Supplementary Table 1).

The relative abundance of *Kribbella* sp. stands out in both samples, having a greater representation in RS. Similarly, the high representation of *Pseudomonas* sp. and *Mesorhizobium* sp. in RS (Figure 2) stands out. Likewise, the presence of strains of the genera *Actinoplanes* sp., *Microcoleus* sp. and *Propionibacterium* sp. seems to be especially abundant in BS (Figure 2).

Viral DNAs have been identified as *Cyclovirus NGChicken15/NGA/2009*, with 18.88% representation in RS. Less abundant in both samples was the mosaic Dasheen virus.

### Potential Functional Analysis

The functional analysis with SUPER-FOCUS present three levels (I-III) and predicted proteins (SEED) with the non-annotated metagenome sequences that allows pool the sequences by potential functional activity clusters. The two metagenomes obtained were assembled using MetaSpades. A total of 813,375 and 676,195 contigs were obtained, respectively.

#### Functional Level I: Large Functional Potential Groups

In the analysis of level I (more general group of potential functional activity), the sequences pooled on the same potential functional activity were ordered according to their relative abundance. In this way, the functional potential content of the samples was ranked, reflecting the abundance of the different subsystems.

Were found 35 functional potential groups (Supplementary Table 2). The most abundant subsystems are those which seem to be related to basal metabolism and basic functions for the survival of bacteria, such as the carbon cycle, amino acid synthesis, and functional activities involved in breathing, among others, while subsystems encompassing more specific characteristics (such as virulence or photosynthesis) are less represented.

It is interesting that the functional potential cluster endowment of “stress response” presents 4.45% RS and 4.36% BS abundances. This functional potential group is among the 10 most abundant, which indicates the high environmental pressure suffered by bacteria in soils contaminated with Hg.

#### Functional Level II: Families of Potential Functional Activities

**FIGURE 3:**
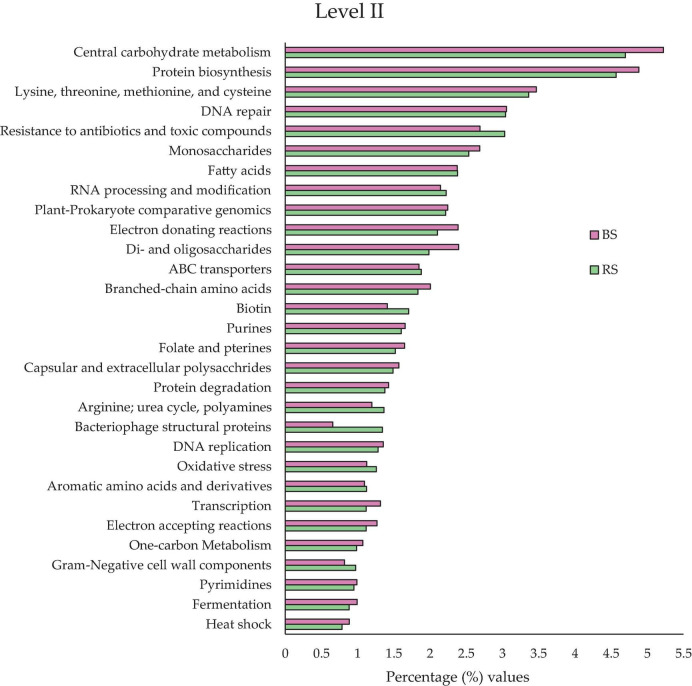
Comparison of the relative abundances of the 30 most abundant functional types in BS (pink bars) and RS (green bars) in level II, all the information at that level could be found in Supplementary Table 3. All the values are expressed in percentages.

At this level (Figure 3), the potential functional activities were collected in a more concrete way, ordering them according to their relative abundances by families of same potential functional activities. Were found at this level 192 functional potential clusters (Supplementary Table 3).

Some potential functional activities are especially relevant and could allow for explaining the biological behavior of the Hg-tolerant edaphic communities. The following activities stand out (Figure 3): “Resistance to antibiotics and toxic compounds” (3.03% RS and 2.96% BS), “ABC type conveyors” (1.88% RS and 1.85% BS) and “oxidative stress” (1.26% RS and 1.13% BS).

#### Functional Level III and SEED: Specific Potential Functional Role and the Protein to Which Sequences Belong

The third level identifies the potential functional role to which the sequences under study belong, revealing the potential function within the metabolism of the bacteria. On level III, 1,186 functional potential groups were found and 24,441 functional potential annotations on SEED were done (Supplementary Tables 4, 5).

The 35 most abundant subsystems belong to a division of the subfamilies found in levels I and II. Furthermore, most of the subfamilies of biological interest in this study seems to be represented with lower relative abundance percentages.

At this level, there are several subsystems that group the resistance to heavy metals and to Hg, in particular. Among them we find various predicted proteins of the *mer* operon, Hg-reductases and ABC-type transporters associated with resistance to heavy metals.

Noteworthy is the large number of subsystems could be linked to resistance to various antibiotics, including beta-lactamases, predicted proteins regulating the BlaR1 family, various proteins of multiple resistance systems, multiple resistance systems linked to the MexAB-OprM and MexEF-OprN complex, fluoroquinolone resistance, vancomycin resistance, mdtABCD flow pump cluster and bacitracin stress response.

There are also subsystems involved in the biological cycle of nitrogen (N). Particularly important are those linked to the nitrogenase complex for atmospheric N fixation. Likewise, the potential functional activities responsible for the promotion of plant growth are represented as “production of auxins,” “metabolism of ethylene,” “siderophore production,” and “phosphate solubilization.” With a major relative abundance in RS.

## Discussion

### Taxonomic Discussion

In the soils analyzed, representatives of six taxonomic phylum appear: Acidobacteria, Actinobacteria, Cyanobacteria, Alphaproteobacteria, Betaproteobacteria, and Gammaproteobacteria, whose analysis and discussion of the findings are described below. The results obtained show how the taxonomical diversity varies between bulk soil and rhizospheric soil, being positive selected by the plant root those that have a potential benefit to the plant, like proteobacteria group (Figure 1 and Table 1) (Moulin et al., 2001; Zai et al., 2021).

**TABLE 1:** General table of bacterial taxonomic identification, together with their relative abundances in BS and RS.

CLASS	ORDER	FAMILY	SPECIES	% RS	% BS	Ref Hg	Ref RS	PGPR	*N*	β-Lac
Acidobacteria	Acidobacterial	Acidobacteriaceae	Species	0	5.29	Vishnivetskaya et al., 2011	Kielak et al., 2016; Xu et al., 2018	–	–	Gonçalves and Santana, 2021
Actinobacteria	Solirubrobacterales	Family	Specie*s*	14.13	32.8	Gołêbiewski et al., 2014	Hernández et al., 2015	–	–	Jauregi et al., 2021
	Propionibacteriales	Nocardioidaceae	*Kribbella* sp.	11.09	7.11	–	Álvarez-Pérez et al., 2017	Shan et al., 2018	Shan et al., 2018; Borah and Thakur, 2020	–
		Propionibacteriaceae	*Propionibacterium acnes*	0	5.42	–	–	–	–	Ramage et al., 2003
			Species	0	6.53	–	Yeager et al., 2017; Armanhi et al., 2018	–	–	–
	Geodermatophilales	Geodermatophilaceae	Species	3.56	7.79	–	Montero-Calasanz et al., 2017	Karray et al., 2020	–	–
	Microccocales	Microbacteriaceae	*Agromyces* sp.	1.01	0	–	Muehe et al., 2015	Lee and Whang, 2020	Hu et al., 2021	Lee et al., 2021
		Cellulomonadaceae	*Cellulomonas* sp.	0.8	0	–	Zhao et al., 2018	Zhao et al., 2021	Suleiman et al., 2019	Zhang et al., 2021
	Micromonosporales	Micromonosporaceae	*Actinoplanes* sp.	0	8.62	–	Gkarmiri et al., 2017	Yamamoto et al., 2018; Kaur et al., 2021	Yamamoto et al., 2018; Kaur et al., 2021	Torres-Bacete et al., 2007
	Actinomycetales	Streptomycetaceae	*Streptomyces chartreusis*	0	1.13	–	Wang et al., 2019	Senges et al., 2018; Vurukonda et al., 2018; Wang et al., 2019	Vurukonda et al., 2018; Wang et al., 2019	–
	Streptosporangials	Thermomonosporaceae	Species	0	8.7	–	Malisorn et al., 2018	–	–	–
Cyanobacteria	Oscillatory	Microcoleaceae	*Microcoleus* sp.	0	5.54	–	Couradeau et al., 2019	Jan et al., 2018	Couradeau et al., 2019	Philippon et al., 2016
Alphaproteobacteria	Rhizobiales	Phyllobacteriaceae	*Mesorhizobium* sp.	4.98	0	Petrus et al., 2015	Garrido-Oter et al., 2018	Menéndez et al., 2020; Alemneh et al., 2021	Garrido-Oter et al., 2018; Menéndez et al., 2020	Rangel et al., 2017
			*Mesorhizobium loti*	6.79	0	–	Garrido-Oter et al., 2018	–	Garrido-Oter et al., 2018	–
		Bradyrhizobiaceae	*Afipia s*p.	3.46	1.94	Petrus et al., 2015	Jaiswal et al., 2017; Zheng et al., 2021	Jaiswal et al., 2017	–	–
			*Rhodopseudomonas* sp.	0	4.7	Deng and Jia, 2011	Wong et al., 2014; Hsu et al., 2021	Wong et al., 2014	Chang et al., 2020	–
			*Rhodopseudomonas palustris*	6.05	0	Deng and Jia, 2011	Wong et al., 2014; Hsu et al., 2021	Wong et al., 2014	Chang et al., 2020	McCully et al., 2018
		Hyphomicrobiaceae	Species	8.88	0	–	Xu et al., 2014	He et al., 2020	Martineau et al., 2014	Zheng et al., 2016
	Caulobacterales	Caulobacteraceae	*Caulobacter* sp.	4.5	2.24	Hu et al., 2005	Gutiérrez-García et al., 2019	Yang et al., 2019	Gutiérrez-García et al., 2019	Escandón-Vargas et al., 2017; Valencia et al., 2020
			*Caulobacter vibrioides*	0.67	0	Wang et al., 2016	–	–	Fatema et al., 2019	Escandón-Vargas et al., 2017
Betaproteobacteria	Burkholderiales	Comamonadaceae	*Variovorax* sp.	5.66	0	–	Belimov et al., 2015; Teijeiro et al., 2020	Toukabri et al., 2021	Woo et al., 2017; Toukabri et al., 2021	Dewi et al., 2020
Gammaproteobacteria	Pseudomonadales	Pseudomonadaceae	*Pseudomonas* sp.	7.9	0	Von Canstein et al., 1999	Zhuang et al., 2021	Zhuang et al., 2021	Geries and Elsadany, 2021	Falodun and Musa, 2020

*Bibliographic reference of the Hg resistance of the taxon (Ref Hg); bibliographic reference of the presence in rhizosphere of the taxon (Ref RS); bibliographic activity reference as PGPR (plant growth promoting rhizobacteria) of the taxon; bibliographic reference of N-binding capacity, and bibliographic reference of the possession of β-lactam resistance genes (β-Lac).*

#### Acidobacteria

Acidobacteria are a phylum within the bacteria domain of species ubiquitous in the soil (Kalam et al., 2020). In the present study, the sample was identified up to the family taxon Acidobacteriaceae (Table 1) (Foesel et al., 2016). In the same way as in our results, the predilection of this bacterial group for the bulk soil has also been described in comparison with the rhizosphere (Kielak et al., 2016; Conradie and Jacobs, 2020). Several authors have also discussed the potential of this bacterial class for the degradation of various pollutants (Feng et al., 2021; Gonçalves and Santana, 2021) and its potential biotechnological application in Hg degradation (Vishnivetskaya et al., 2011; McDaniel et al., 2020).

#### Actinobacteria

Actinobacteria are a phylum and class of Gram-positive bacteria and are the group with the highest representation in BS and the second most represented in RS. This taxon is particularly interesting because of its wide biotechnological potential. Most antibiotics and many of the compounds used in the production of medicines come from the species in this class (Hopwood, 2007). There are recent studies of the relationship of these bacteria with resistance to various heavy substances (Yun et al., 2020), among which are the mer genes of resistance to Hg (Christakis et al., 2021). In this manuscript, representatives have been found in seven of the orders pertaining to this class (Table 1).

Two families and two species of the Propionibacteriales order were identified. *Propionibacterium acnes* is first described as Hg resistant as well as its presence in rhizospheric soils.

*Kribbella* sp. is linked to both types of soil. Its resistance to Hg is not included in the scientific literature, although some authors have described strains of this genus resistant to other heavy metal divalent cations like Cu, Ni, and Cd (Chanda et al., 2017; Rosenfeld et al., 2018). Other authors describe the capacity of produce siderophores by some species (Acquah et al., 2020). The presence of this bacteria in a Hg-contaminated soil and the data found on the bibliography suggests postulating that species as a good target to look for strains with possible biotechnological potential for use in soil bioremediation.

Within the Geodermatophilales order, the Geodermatophilaceae family present in both types of soils was identified (Table 1). Although bacteria associated with the rhizosphere of some plant species have been described in the literature (Montero-Calasanz et al., 2017), it was found with a greater abundance in BS in the present study. There are references to some species in this family as resistant to heavy metals (Kou et al., 2018), although no evidence of their description as resistant to Hg has been found.

In the order of Microccocales, two families and two species were detected (Table 1), *Agromyces* sp. and *Cellulomonas* sp. Neither of these two species is cited as resistant to Hg, a fact that is first described in this study. Its resistance to other heavy metals is described (Corretto et al., 2017; Brookshier et al., 2018). The presence of *Agromyces* sp. associated with *Arabidopsis halleri* roots in the extraction of heavy metals by phytoextraction is not known (Muehe et al., 2015); the role of *Cellulomonas* sp. in plant rhizome as a producer of IAA is known (Zhao et al., 2018). The findings on these two bacteria are postulated by organisms with a possible biotechnological potential to look for strains for use in soil bioremediation in this study, despite their low representation in the data obtained (Table 1).

In the Micromonosporal order, a single species *Actinoplanes* sp. was detected, only in BS, being described as good PGPR (Table 1) (Gkarmiri et al., 2017; Yamamoto et al., 2018). Works such as that of Wang et al. (2019) have demonstrated the tolerance to various heavy metals by *Actinoplanes* sp.; no resistance references to Hg were found. The resistance to Hg of these species it’s first described in this study.

The genus *Streptomyces* (Actinomicetales) is one of the most studied due to its high biotechnological and industrial potential, since a large quantity of antibiotics currently used clinically are derived from these bacteria (Hopwood, 2007). Its resistance to Hg has been referenced for more than 20 years (Ravel et al., 1998). *Streptomyces chartreusis* has been described as good PGPR (Table 1) (Senges et al., 2018; Vurukonda et al., 2018; Wang et al., 2019). This is the first time *S. chartreusis* has been referred to in a soil contaminated with Hg. The potential PGPR and its remediation capability noted in the literature, along with its presence in the study soil, suggests *S. chartreusis* as a good target for search strains that could be used in the remediation of this environment.

#### Cyanobacteria

Cyanobacteria (bacteria domain) include bacteria capable of performing oxygenic photosynthesis. It’s a phylum that have an extensive ecological distribution, as well it’s needed to take in account that the plots sampled are located in rainwater leaching areas and close to water sources, so it understandable to find this phylum on the sample (Mehetre et al., 2022). The species *Microcoleus* sp. was detected (Table 1). *Microcoleus* sp. has not been described as resistant to Hg. Godoy-Lozano et al. (2018) shows the high potential of this species to degrade various heavy metals. The data collected in the literature and their presence in these soils are indicative of their potential as research targets for its possible use in the bioremediation of Hg.

#### Alphaproteobacteria

*Mesorhizobium* sp. and *Mesorhizobium loti* (Phyllobacteriaceae) have been studied for their ability to form nodules in plant roots and fix N (Garrido-Oter et al., 2018). They are of great interest for their close relationship with the plant root and have been described as resistant to various heavy metals, such as Cd or Pb (Fan et al., 2018), highlighting their strong potential for use in soil remediation.

*Afipia* sp., *Rhodopseudomonas* sp. and *Rhodopseudomonas palustris* (Bradyrhizobiaceae): *Afipia* sp. arouses interest for its potential use in remediation of soils contaminated with Hg (Petrus et al., 2015). *Rhodopseudomonas* sp. and *Rhodopseudomonas palustris* are described as PGPR (Wong et al., 2014; Hsu et al., 2021). Some authors (Batool and Rehman, 2017; Xiao et al., 2021) relate the PGPR capacity of *Rhodopseudomonas* sp. and *R. palustris* to their ability to remediate Hg-contaminated soils.

*Calubacter* sp. and *Caulobacter vibrioides* (Caulobacterales) have been described as resistant to various heavy metals because they host divalent cation ATPase transporters on their membranes (Hu et al., 2005). As for *C. vibrioides*, it has solubilizing activity of selenium used in processes of detoxification of Hg (Wang et al., 2016). As well, this bacterias appear in the bibliography described as nitrogen fixers (Gutiérrez-García et al., 2019). For these reasons, these bacteria have a biotechnological interest for use in future bioremediation pathways.

#### Betaproteobacteria

For this Class, only one specie could be identified. *Variovorax* sp. (Burkholderiales) has been characterized by Belimov et al. (2015), noted for its activity as PGPR and its resistance to various heavy metals, although not appearing in the literature as resistant to Hg. Having found this species in RS with high concentration of Hg postulates it as a good candidate to further studies to find strains from this species with potential use in bioremediation.

#### Gammaproteobacteria

Gammaproteobacteria are a diverse class of Gram-negative bacteria with a wide biological distribution. *Pseudomonas* sp. have a high biotechnological interest and have been studied for their characteristics as PGPR (Yasmeen et al., 2021). The bioremediation capacity of Hg is well known (González et al., 2021; Imron et al., 2021; Robas et al., 2021a). Therefore, a study of its potential use in the remediation of contaminated soils and look for non-phytopathogenic specific strains is of interest.

#### Viruses

Two viral families were identified in the metagenome, Circoviridae and Potyviridae. The first is a family that is distributed in the environment infecting mainly vertebrates (Delwart and Li, 2012). Only the species Cyclovirus NGChicken15/NGA/2009was identified, it is a virus typically pathogenic from farm birds. It is interesting and remarkable that of the 21% of the relative taxonomic abundance of viruses in the RS sample, 18.88% belong to a single virus that is very far from its natural host. Due to the ubiquity of this virus and its presence in our sample only in RS we suspect that it could be a virus very similar to NGchicken15 that is infecting the plant or any of the bacteria in the rhizosphere such as prophage.

From the family Potyviridae was identified the species Dasheen mosaic virus, very common species pathogenic of vegetables (Francki et al., 2012) with a similar relative abundance in both soils (1.66% RS and 2.19% BS) It is not strange to find this species both in the fraction of SL and RF given that the samples were collected at a time of high growth and therefore of a greater transmissibility of this virus to plants and to the soils.

### Functional Analysis

Functional potential bioinformatic analysis allows the identification of potential metabolic activities and processes. In this way, it is possible to sort the potential functions of the microbial community according to their abundance. However, minority activities should be taken into account as long as they allow the biological behavior of these communities to be interpreted. In this sense, most of the functional potential clusters identified, and sequences associated to a protein belong to basal metabolism. However, the mechanisms of Hg resistance, those involved in oxidative stress, N metabolism and PGPR activity are more important from an ecological and functional potential point of view. In addition, sequences associated to a protein have been found whose presence in soils can only be interpreted as indicating biological contamination (Li et al., 2020; Stange and Tiehm, 2020). Since there is no antibiotic pressure on the analyzed soils, the mechanisms of resistance commonly described in clinical studies should not be detected in environmental samples. For this reason, in the area of “One Health” they deserve to be analyzed and interpreted as bioindicators of biological pollution. Microbial soil communities can act as reservoirs from which information can be transferred horizontally to potential pathogens, becoming a threat to human, animal and environmental health (Wang et al., 2021).

#### Antibiotic Resistance

Soils, especially those under high environmental pressure, act as a natural reservoir of resistance to existing antibiotics or provide the potential to host bacteria of clinical importance (Yan et al., 2020; Liao et al., 2021). Several studies show the existence of a co-choice between the presence of various toxic compounds in the environment and the selection of antibiotic resistance naturally, together with co-resistance to heavy metals and antibiotics (Yan et al., 2020; Mazhar et al., 2021; Robas et al., 2021b).

At level II this functional cluster were more represented in RS than in BS. At level III of the metagenome study, several specific role clusters for resistance to rare antibiotics in the natural environment have been identified (Supplementary Table 12), such as β-lactamases and mechanisms associated with resistance to beta-lactams and predicted proteins associated with gene responses to these antibiotics BlaR1 and MecR1 (Silveira et al., 2018).

Likewise, the presence of various transporters dependent on ATP of Pb, Cb, Cu, and Hg in the group of functional activities regulating the beta-lactams BlaR1 was found, and a direct relationship between the presence of these heavy metals and β-lactam resistance. Among the predicted proteins related with β-lactam resistance genes isolated in this study, some belonging to classes A, C, and D were identified.

Class A includes several subsystems, all fundamentally linked to ampicillin resistance, that are rarely used clinically owing to numerous described resistances that exist for this antibiotic (Kaye et al., 2000; Rice et al., 2001). CTX-M-16 was found, which gives greater catalytic power than other cefotaximes (Bonnet et al., 2001). The finding in the present work of resistance mechanisms to these antibiotics was evidence of the selection of resistance mechanisms of clinical origin, especially when occurring in semi-synthetic compounds that do not occur in nature.

The AmpC-type β-lactamases are of great clinical importance because they are hydrolyzed penicillins, cefamycins, oxyminocephalosporins and monobactams, although they are not active against fourth-generation carbapenemic cephalosporins (Jacoby, 2009; Aguirre et al., 2020). The relevance of these genes is provided by their high transmissibility, since many are found in plasmids (Ku et al., 2019; Rensing et al., 2019).

The MexAB-OprM and MexEF-OprN complexes are a protein assembly of membrane transporters that provide multi-resistance to antibiotics, identified primarily in *Pseudomonas* sp. and are highly linked to multi-resistance in *P. aeruginosa* (Ma et al., 2021). As shown in Table 1, there is high probability that these genes that could confer multi-resistance can be associated with the *Pseudomonas* identified in this manuscript. In the analysis of the sequences, were predicted proteins from the MexT regulator was found, which is inactivated by some Hg resistance genes, making the strains carrying the MexEF-OprN complex sensitive to carbapenems (Köhler et al., 1999).

At level III, sequences related to RND transporters have been found in multi-resistance efflux pumps functional potential cluster. Among these, there is a large representation of MexAB-OprM and MexEF-OprN, along with MexCD-OprJ antibiotic transporters, related not only to antibiotic resistance but also mediate processes of quorum sensing (Alcalde-Rico et al., 2018). Other antibiotic resistance predicted proteins found were those from AcrAB-TolC, a system responsible for the expulsion of antibiotics, such as penicillin G, cloxacillin, naphthyllin, macrolides, novobiocin, linezolid, and fusidic acid derivatives; this system is commonly associated with E. coli (Anes et al., 2015; Byrd et al., 2021). Proteins from MdtABC genes have also been predicted in SEED, related TolC, which give resistance to novobiocin, quinolones and phosphomycin, among others (Anes et al., 2015). CmeABC membrane transporters were also found, related to Campylobacter jejuni’s resistance to a wide variety of antibiotics (Lin et al., 2002; Yan et al., 2006), although authors such as Stopnisek et al. (2016) have reported the presence of these genes in other species within the Rhizobiales order.

Some proteins directly related to antibiotic multi-resistance mechanisms have been predicted with the metagenome sequences in Gram-positive bacteria cluster. The MdtRP operon of *Bacillus* sp. confers resistance to several antibiotics such as novobiocin, streptomycin and actinomycin, and is regulated by the MarR repressor (Gupta et al., 2019; Warmbold et al., 2020).

Another of the functional subsystems identified in level III and SEED is related to tripartite protein expulsion systems in Gram-negative bacteria. Similar to the already noted MexAB-OprM or AcrAB-TolC, they belong to membrane transporters RND (Daury et al., 2016). No specific identification of any of these sequences was achieved beyond identifying them as antibiotic ejection systems within the RND group and related to tripartite proteins such as MdtABC, and ejection proteins from genes such as TolC or OprN. This highlights a variety of resistance systems that bacteria can possess in a hostile environment, and which are still unknown.

An important functional cluster related to fluoroquinolone resistance has been found. These are synthesis compounds, not found naturally in environmental samples. Hooper (2000) describes how fluoroquinolone resistances are acquired by mutations in the genes of Topoisomerases II and IV, predicted proteins present in the metagenome sequences of the samples analyzed at level III and SEED. Similarly, pumps from Lde genes were found, which are specific to fluoroquinolones (Godreuil et al., 2003).

A functional cluster and predicted proteins related with vancomycin resistance were also found. The proteins predicted VanA, VanB, VanH, VanR, VanS, VanW, VanX and VanZ, all of which were directly related to vancomycin resistance (Qureshi et al., 2014; Stogios and Savchenko, 2020) and VanZ, which in turn gave teicoplanin resistance (Qureshi et al., 2014) and VanW whose role in resistance mechanisms is still unknown. These genes are usually grouped by their function and level of resistance, thus taking the VanAB, VanHAX, and VanRS groups (Bugg et al., 1991; Arthur et al., 1992). The presence of some sequences that predict proteins, such as those related with VanAB genes, appearing in plasmids has been studied (Ishihara et al., 2013; Qureshi et al., 2014; Sivertsen et al., 2016), giving this resistance greater potential to be transmitted to other species. Several proteins related with that regulation of the activity of this resistance were also found, such as VanRS and a histidin-kinase system that activates the resistance system (Arthur and Quintiliani, 2001; Qureshi et al., 2014; Stogios and Savchenko, 2020).

Another group of resistance functional potential cluster was that corresponding to bacitracin resistance. The predicted proteins in SEED correspond to ABC flow pumps associated with the *Bacillus* genus such as BceAB, BceR, YvcPR, YxdM, YclH, YknY, BseL and LiaRS (Mascher et al., 2004; Kingston et al., 2014). Some of these genes have also been reported in other Gram-positives, such as some species of *Enterococcus* and *Clostridium* (Charlebois et al., 2012; Zhou et al., 2019).

#### Hg Resistance and Oxidative Stress Response

The mechanisms of resistance to Hg are widely described in the microbial world. For this reason, it is not uncommon to detect various predicted proteins associated with genes from operon *mer* (MerC, MerE, and MerT) (Gionfriddo et al., 2020) and mercuroreductases in the samples analyzed (Supplementary Table 6). In the same way, some resistance and transport mechanisms for divalent cations (Co, Zn, and Cd primarily) (Supplementary Table 6) were found, revealing the probably participation of that mechanisms in the resistance to Hg of the microorganisms. Likewise, ABC-type transporters (ATP-binding cassette), capable of providing resistance to bacteria against various toxins (Acar et al., 2020; Thomas and Tampé, 2020), account for almost 50% of membrane transporters in level II. At SEED level it can be found some ABC and RND efflux systems related with resistance with divalent cations and heavy metals (Supplementary Table 6). These transporters are widely distributed throughout the metagenome and primarily associated with resistance systems, acting as efflux pumps for different toxic compounds (Supplementary Table 3).

Environmental factors are known to cause oxidative stress to the colonizing microorganisms of contaminated soils. In order to colonize these environments, bacteria need effective biological mechanisms. Stress response genes give microorganism methods of adaptability to host situations and environments that may affect their normal development (Chakraborty and Kenney, 2018; Dweba et al., 2018; Kokou et al., 2018). These potential functional activities are found with a high relative abundance (Supplementary Table 7), as the soil is a “nutritional desert” and has the abiotic stress of high Hg concentration. These factors are in a similar proportion in both samples; it seems that BS and RS exert a different environmental pressure on organisms in terms of stress. Various ABC transport mechanisms have been developed, which function as part of the stress response machine in a hostile environment (Teichmann et al., 2018; Van Goethem et al., 2018). As well some sequences related with the carbon starvation, like proteins of Slp (Alexander and St John, 1994) and Sspa (Yin et al., 2021) were found. And functional potential clusters related with weather conditions (Cold and heat shock, desiccation stress and osmotic stress) (Supplementary Table 7).

#### N Metabolism and PGPR

N is a limiting macronutrient for the proliferation of microorganisms and the growth of plants. Therefore, the enzymes involved in the synthesis of nitrogen compounds usable by plants are relevant. In the present study, in level I was found a functional potential cluster that involves the N metabolism (Supplementary Table 8). Several sequences have been associated with proteins related to the assimilation of nitrate and nitrite as the Nar (NarA, NarD, NarE, NarK, NarL, and NarP) (Fukuda et al., 2015). Among the N metabolism, proteins from Nir denitrification genes were identified. Some of these sequences, related with genes such as NirT and Nos, are involved in N monoxide denitrification and formation (Bergaust et al., 2012; Belbahri et al., 2017). At level III, was alsa found a cluster related with the nitrogen fixation, fundamentally from the operon Nif were identified, which codes for the nitrogenase complex, fundamental in the fixation of atmospheric N in the soil (Di Cesare et al., 2018; Dasgupta et al., 2021; Supplementary Table 8).

Plant growth-promoting bacteria are characterized by their ability to produce and/or adapt to a hostile environment, such as auxin production, phosphate solubilization, ACC degradation, and siderophore production. Several sequences clustered in potential functional activities involved in the synthesis of auxins, an important factor promoting the growth of plants, have been identified (Mishra et al., 2021). Specifically, 3-indolacetic acid (IAA) is related to a large number of processes that improve plant quality. Some proteins from the principal biosynthetic routes of IAA, indole-3-pyruvate and indole-3-acetamide were predicted (Nascimento et al., 2021), such as proteins IorA and IorB (Supplementary Table 9). Likewise, IAA has the capacity to improve the tolerance of plants to adverse conditions and stress by heavy metals (Ma et al., 2011; Nazli et al., 2021). Some authors (Ma et al., 2011; Zainab et al., 2020) have established a relationship between rhizospheric bacteria producing IAA and significant improvement together with greater speed in phytoextraction of heavy substances and recovery of contaminated soils.

Plants and microorganisms compete for phosphorus present in the environment; therefore, the solubilization of phosphorus by microorganisms contributes to the promotion of plant growth (Pereira et al., 2020). A large number of proteins genes have been predicted for acid phosphatases, phytases, gluconate dehydrogenases, ketogluconate dehydrogenases, and glucose-1-dehydrogenase (Supplementary Table 10), which are encompassed in the context of phosphate solubilization (Suleman et al., 2018).

The predicted proteins of the AcdS genes of the ACC deaminase (1-aminocyclopropane-1-carboxylate desaminase) found in our metagenome interfere with the synthesis of ethylene in the plant by degradation of a metabolic precursor, thereby reducing stress in the tissues (Glick, 2014; del Carmen Orozco-Mosqueda et al., 2020). Ethylene is a marker of plant stress and senescence, so this enzyme helps plants withstand stressful environments, such as soils contaminated with heavy metals.

A wide variety of siderophore functional potential clusters were also identified (Supplementary Table 11), it can be found at level II a functional potential cluster related with siderophores (Supplementary Table 3). Siderophores act as metal chelators favoring, among other potential functions, the absorption of iron and its entry into the food chain. Plants that are able to use bacterial siderophores as a source of iron (Wang et al., 1993) increase their chances of survival and adaptation to contaminated environments.

## Conclusion

Several conclusions can be drawn from this study.

In the taxonomic analyze, the most abundant microbial genome analyzed belongs to the bacteria domain. The prevalent taxa are those of Actinobateria and Alphaproteobacteria. Betaproteobacteria and Gammaproteobacteria seems to be intimately linked to rhizospheric soil (RS). Likewise, Cyanobacteria and Acidobacteria only have representation in bulk soil (BS). Similarly, the genome belonging to the Domain Eukarya involved in the potential functional activity of microbial activity was detected. The viral genomes present in the sample are interpreted as prophages.

On the functional potential profiling, the presence of antibiotic resistance mechanisms and other toxic compounds could confirm previous studies pointing to their co-selection in Hg-contaminated environments. The finding of resistance mechanisms proper to human clinical, evidence of biological contamination, suggests that these environments may behave as reservoirs. Although, the presence of Hg resistance functional clusters and involved in the response to oxidative stress are present as a minority; however, their biological significance justifies the behavior of the microbial community. The abundance of PGP and N-fixation functional activities detected in the metagenome sequences may be an opportunity for further selection of both effective bioremediation strains and genes for promoting biotechnological use in the production of GMOs.

## Data Availability Statement

The original contributions presented in the study are included in the article/Supplementary Material, further inquiries can be directed to the corresponding author.

## Author Contributions

AP and PJ supervised the project and acquired funding for this research. All authors designed the experiments, made intellectual contributions, conducted the experiments, analyzed the data, wrote and edited the manuscript.

## Conflict of Interest

The authors declare that the research was conducted in the absence of any commercial or financial relationships that could be construed as a potential conflict of interest.

## Publisher’s Note

All claims expressed in this article are solely those of the authors and do not necessarily represent those of their affiliated organizations, or those of the publisher, the editors and the reviewers. Any product that may be evaluated in this article, or claim that may be made by its manufacturer, is not guaranteed or endorsed by the publisher.

## Supplementary Material

Supplementary Table 1 Relative abundances at all taxonomical levels (Metaphlan data table).

Supplementary Table 2 Relative abundances of SUPERFOCUS functional level I.

Supplementary Table 3 Relative abundances of SUPERFOCUS functional level II.

Supplementary Table 4 Relative abundances of SUPERFOCUS functional level III.

Supplementary Table 5 Relative abundances of SUPERFOCUS at SEED function level.

Supplementary Table 6 Relative abundances of SUPERFOCUS functional levels I–III and SEED of resistance to heavy metals potential functional activities.

Supplementary Table 7 Relative abundances of SUPERFOCUS functional levels I–III and SEED of stress response and oxidative stress potential functional activities.

Supplementary Table 8 Relative abundances of SUPERFOCUS functional levels I–III and SEED of nitrogen metabolism potential functional activities.

Supplementary Table 9 Relative abundances of SUPERFOCUS functional levels I–III and SEED of IAA potential functional activities.

Supplementary Table 10 Relative abundances of SUPERFOCUS functional levels I–III and SEED of ACCd potential functional activities.

Supplementary Table 11 Relative abundances of SUPERFOCUS functional levels I–III and SEED of siderophores potential functional activities.

Supplementary Table 12 Relative abundances of SUPERFOCUS functional levels I–III and SEED of resistance to antibiotics potential functional activities.
